# In Vitro and In Vivo Evaluation of Composite Oral Fast Disintegrating Film: An Innovative Strategy for the Codelivery of Ranitidine HCl and Flurbiprofen

**DOI:** 10.3390/pharmaceutics15071987

**Published:** 2023-07-20

**Authors:** Aisha Rashid, Syed Haroon Khalid, Muhammad Irfan, Sajid Asghar, Waleed Y. Rizg, Fahad Y. Sabei, Eman Alfayez, Hanaa Alkharobi, Awaji Y. Safhi, Khaled M. Hosny, Muhammad Sohail Arshad, Ikram Ullah Khan

**Affiliations:** 1 Department of Pharmaceutics, Faculty of Pharmaceutical Sciences, Government College University Faisalabad, Faisalabad 38000, Pakistan; draisharashid@gmail.com (A.R.); haroonkhalid80@gmail.com (S.H.K.); manipharma1@gmail.com (M.I.); sajuhappa@gmail.com (S.A.); 2 Center of Innovation in Personalized Medicine (CIPM), 3D Bioprinting Unit, King Abdulaziz University, Jeddah 21589, Saudi Arabia; wrizq@kau.edu.sa; 3 Department of Pharmaceutics, Faculty of Pharmacy, King Abdulaziz University, Jeddah 21589, Saudi Arabia; kmhomar@kau.edu.sa; 4 Department of Pharmaceutics, College of Pharmacy, Jazan University, Jazan 45142, Saudi Arabia; fsabei@jazanu.edu.sa (F.Y.S.); asafhi@jazanu.edu.sa (A.Y.S.); 5 Department of Oral Biology, Faculty of Dentistry, King Abdulaziz University, Jeddah 80209, Saudi Arabia; ealfayez@kau.edu.sa (E.A.); halkharobi@kau.edu.sa (H.A.); 6 Department of Pharmaceutics, Faculty of Pharmacy, Bahauddin Zakariya University, Multan 60800, Pakistan; sohailarshad@bzu.edu.pk

**Keywords:** Lycoat^®^ RS780, flurbiprofen, ranitidine hydrochloride, composite ODF, microparticles

## Abstract

Here, we evaluate the feasibility of co-loading plain ranitidine hydrochloride (RHCl) and microencapsulated flurbiprofen (FBP) in a Lycoat^®^ RS780-based oral fast disintegrating film (ODF). These films were developed by the solvent casting method to minimize the adverse effects of FBP and reduce the dosage form burden on patients. Optimized FBP microparticles (M3) with an average size of 21.2 ± 9.2 µm were loaded alone (F1) and in combination with plain RHCl (F2) in the composite ODF. All films were evaluated physicomechanically and physicochemically. These films were resilient, flexible, and disintegrated within thirty seconds. SEM images showed intact FBP microparticles in both formulations and, moreover, did not observe an interaction between the drug and film components. Microencapsulated FBP was released in a controlled manner over 48 h from the proposed formulations, while RHCl was released within 5 min from F2. After in vitro evaluation, formulations were also tested for in vivo anti-inflammatory activity, cytokine (TNF-α and IL-6) levels, and gastroprotective effects in rats. The anti-inflammatory activity and gastroprotective effect of F2 were markedly higher than pure FBP and other synthesized formulations (M3 and F1). The average score of gastric lesions was in the order of pure FBP (15.5 ± 1.32) > M3 (8 ± 2) > F1 (1 ± 0.5) > F2 (0.5 ± 0) > control (0). Additionally, F2 showed a sustained anti-inflammatory effect up to 10 h in the rat paw edema model. Furthermore, F2 also markedly reduced TNF-α and IL-6 levels. Conclusively, the Lycoat^®^ RS780-based composite film could be a promising carrier for the co-loading of microencapsulated FBP with RHCl. In the future, an optimized formulation (F2) could be capable of countering the issues related to multiple drug administration in geriatric patients and evading the gastric irritation associated with FBP.

## 1. Introduction

The oral administration of drugs serves as an ideal delivery route by offering ease of intake, patient compliance, and safety. However, this route also proposes certain limitations, e.g., some drugs undergo extensive hepatic metabolism, and hydrophobic drugs show an inappropriate dissolution and poor absorption. Ultimately, both types of drugs show poor bioavailability. On the other hand, drugs with a short half-life can be taken in multiple doses with a marked variation in peak plasma levels and thus culminate in undesirable effects such as ulcers [1]. To overcome these aforementioned shortcomings, pharmaceutical scientists have proposed various approaches such as solid dispersion, salt formation, microparticles, nanoparticles, cyclodextrin-based inclusion complexes, and oral fast-disintegrating tablets and films [2,3,4,5].

Among these approaches, ODF has emerged as a leading carrier for the oral delivery of hydrophilic and hydrophobic drugs. ODF offers several advantages over conventional oral dosage forms, such as rapid disintegration without mastication or water intake, the immediate onset of action, no risk of choking, improved patient compliance, the avoidance of a harsh intestinal environment, evading first-past metabolism, while being cost-effective as well. Moreover, ODF are equally suitable for geriatric, pediatric, bedridden, mentally ill, nauseous patients, and others suffering from oral pain due to mucositis, periodontal diseases, or after oral surgical procedures. The ODF also presents relatively more accurate dosing compared to syrups or drops [6,7]. In recent times, the composite ODF carrying microparticles, nanoparticles, solid dispersion, and self-emulsifying systems [8,9,10] were developed to control the drug release rate and site-specific delivery to enhance the bioavailability of drugs.

FBP is a nonsteroidal anti-inflammatory drug (NSAID) that is used to relieve pain, swelling, and joint stiffness caused by osteoarthritis and rheumatoid arthritis. It is also used to treat mild to moderate dental pain, menstrual cramps, and muscle aches. It is a hydrophobic molecule with a short half-life (3–4 h) and undergoes hepatic metabolism. A frequent dosage is required due to its short-half life, which ultimately causes unwanted side effects such as gastric irritation and ulcerative lesions [11]. Therefore, RHCl (H_2_ receptor antagonist) is frequently recommended as a supportive treatment with FBP [12]. RHCl is a hydrophilic molecule that reduces acid secretion and lowers the risk of bleeding and ulceration. After oral administration, RHCl has also shown low bioavailability, i.e., 50%, due to hepatic metabolism and microbial degradation [13]. As this combination is frequently used in geriatric patients, combining them in a single formulation could be beneficial. For example, bilayer tablets containing RHCL in their immediate layer and FLB in a sustained layer were developed [14,15]; however, these tablets could pose challenges in geriatric patients. However, this combination has never been encapsulated in ODF. 

Lycoat^®^ is pregelatinized by a hydroxypropyl pea starch-based polymer and is available in different grades (Lycoat^®^ NG 73, RS720, and RS780) [16,17,18]. Lycoat^®^ is a highly water soluble and non-gelling polymer that provides good mechanical properties, fast disintegration, and dissolution to the formulation [19] with a pleasant feel in the mouth [20]. Ethyl cellulose (EC) is a lipophilic and biodegradable polymer. It is insoluble in all physiological pH but erodes and swells at different pH conditions. EC is extensively employed to prepare a sustained-release microparticle-based system [21,22,23,24]. 

To circumvent the aforementioned pitfalls, the novel composite ODF of Lycoat^®^ RS780 was developed and loaded with two drugs for the very first time, as per our knowledge. FBP was loaded in EC-based microparticles (MPs) to provide controlled drug release and augment its bioavailability [25]. Plain RHCl was loaded in ODF to provide rapid absorption from oral mucosa and prevent gastric irritation of the mucosa and ulcer, as observed with FBP during its prolonged use in patients suffering from arthritis. Optimized formulations were evaluated by in vitro and in vivo testing. 

## 2. Material and Methods

### 2.1. Material

Flurbiprofen (FBP) and ranitidine HCl (RHCl) was obtained from Axis Pharmaceuticals (Pakistan). Lycoat^®^ RS780 and Pearlitol Flash^®^ (Mannitol Starch) were received as gift samples from Roquette Frères (France). Polyvinyl alcohol (PVA 1500), polyethylene glycol (PEG 400), propylene glycol (PG), and dichloromethane (DCM) were obtained from Daejung Chemicals and Metals Co., Ltd. (Siheung, Republic of Korea). Glycerin (GLY) was obtained from Sigma-Aldrich (Darmstadt, Germany). All other excipients and chemicals were of an analytical grade.

### 2.2. Methods

#### 2.2.1. Fabrication of FBP Microparticles (MPs)

**Figure 1:**
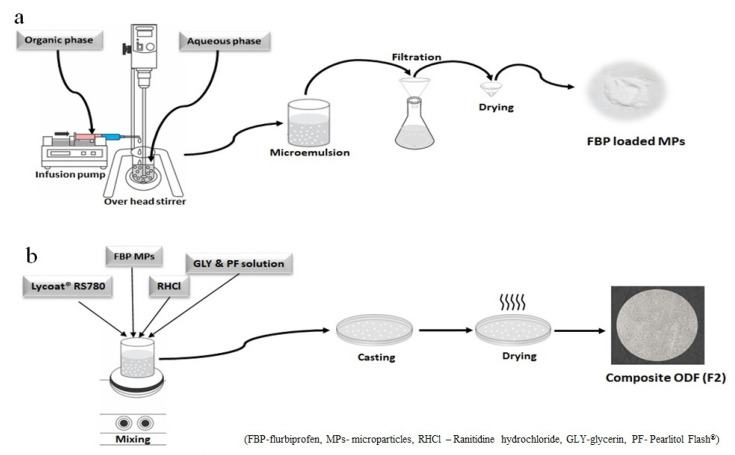
Schematic representation for the development of (**a**) Microparticles and (**b**) Composite ODF.

MPs were prepared using a solvent evaporation technique with the minor modification of a previously reported method [25] and using the ingredients mentioned in Table 1. Briefly, for harvesting MPs, an organic phase containing the drug and EC was injected into an aqueous phase with constant stirring (Figure 1a). To obtain uniform-size particles, the organic phase was injected dropwise via a 25-gage syringe, and its flow rate was maintained at 100 mL/hour through an infusion pump. The given solution was stirred (SCILOGEX OS40-pro LCD digital overhead stirrer) at 1500 rpm for 1 h to remove DCM. Afterward, MPs were collected on Whatman Filter paper # 40 and washed with distilled water and dried overnight at room temperature. All formulations were fabricated in triplicate and dried MPs were kept in airtight vials for future use. 

#### 2.2.2. Screening for Optimized Blank ODF

Initially, various plasticizers (PG, PEG 400, and GLY) were evaluated at different concentrations (1, 5, 10, 15% *w/w* of polymer) with the constant concentration of Lycoat^®^ RS780 (10% *w/v*) (Table 2). After the selection of an optimum plasticizer, different concentrations of Lycoat^®^ RS780 (1, 5, 10, 15, and 20% *w/v*) were screened to select an adequate concentration of the polymer (Table 3). These films were prepared by the solvent casting method as described by Alayoubi, et al. [26] with a slight modification. Initially, a solution of Lycoat^®^ RS780 and Pearlitol Flash^®^ (PF) was prepared in distilled water with constant stirring at 500 rpm (AM4, Multiple Heating Magnetic Stirrer, VELP Scientifica, Italy) and 50 °C for 1 h. After cooling, a plasticizer solution was added to the polymeric solution and stirred for 30 min. The resultant solution was cast in the Petri dish and dried overnight at room temperature. The dried film was carefully removed from the Petri dish and cut into 3 × 2 cm^2^ stamp-shaped films. These film strips were wrapped in aluminum foil and stored in a desiccator until further use. Afterward, the obtained films were evaluated for surface imperfection, appearance (transparent or translucent), consistency, stickiness, peelability, flexibility, and in vitro disintegration (DT).

#### 2.2.3. Fabrication of Composite ODF

The FBP MPs loaded composite ODF was developed according to a procedure reported by Lai, et al. [28] with a minor modification. Optimized MPs (M3) were dispersed in the aqueous solution of an optimized Lycaot^®^ RS780 formulation (L15) and were referred to as F1 (Table 4). Then, this solution was cast in the Petri dish and dried overnight at room temperature. The resultant film was carefully removed from the Petri dish and cut into a suitable size (stamp-shaped film 6 cm^2^) (Figure 1b). These films were wrapped in aluminum foil and stored in a desiccator until further analysis. 

##### Incorporation of Dual Drug in Composite ODF

The dual drug-loaded composite ODF (F2) was also developed by the solvent casting method, as described above. Briefly, RHCl was initially dissolved in an optimized Lycaot^®^ RS780 formulation (L15), and later on, the designated quantity of MPs (M3) was added to the above solution (Table 4). This suspension was mixed well and cast in the Petri dish. The whole experiment was accomplished in darkness. After drying, the film was cut into a suitable size and wrapped in aluminum foil until further testing. 

#### 2.2.4. Characterization of MPs

##### Rheological Studies of MPs

The prepared MPs (M3) powder was subjected to rheological studies to evaluate the flow properties [25].

Bulk and tapped densities were measured to calculate the compressibility index (carr’s index (Ci)) and Hausner’s ratio.

$$Bulk\;Density=\frac{Weight\;of\;MPs}{Initial\;volume\;of\;MPs}$$

$$Tapped\;Density=\frac{Weight\;of\;MPs}{Volume\;of\;MPs\;after\;100\;tapings}$$

$$Ci(\%)=\frac{Tapped\;density-Bulk\;density}{Tapped\;density}\times 100$$

The particles showed good flow characteristics if Ci was less than 15%, while above 25% reflected poor flow characteristics.

Hausner’s ratio is another index for measuring the flowability of microparticles and can be calculated by the following equation:

$$Hausner’s\;Ratio=\frac{Tapped\;density}{Bulk\;density}$$

If its value is less than 1.2, then particles are preferably free-flowing. However, Hausner’s ratio close to 1 specified good flow properties.

The angle of repose was calculated by passing microparticles through the funnel on a horizontal surface. The height (*h*) of the heap and radius (*r*) of the cone base was calculated. Afterward, the angle of repose (*θ*) was calculated by the following equation:

$$Tan\theta =\frac{h}{r}$$

An angle less than 40° reflected free flow properties, while an angle between 25 and 30° specified excellent flow properties.

##### Solid State Characterization of MPs

The surface topology of FBP and the optimum formulation (M3) were observed by a scanning electron microscope (SEM) (Cube series, EMCRAFTS, Sungdong-gu, Seoul, Republic of Korea). The drug-polymer compatibility was studied by Fourier transform infrared spectroscopy (FTIR, Agilent Technologies, Waldbronn, Germany). An assessment of the crystallinity or amorphicity of the drug and formulation was accomplished by X-ray diffractometry (XRD, D8 ADVANCE, Bruker, Karlsruhe, Germany). The effect of thermal changes on the sample weight was investigated by differential scanning calorimetry and thermogravimetric analysis (DSC-TGA, SDT Q600, New Castle, DE, USA) [29].

##### Drug Content in MPs

In total, 20 mg of MPs containing the drug were dissolved in 10 mL of DCM and then diluted with a phosphate-buffered solution (PBS) pH 6.8. DCM was removed by the vigorous agitation of the solution for 1 h, and the polymer was removed using a 0.45 µm syringe filter. After filtration, the absorbance of flurbiprofen was measured by a UV-Visible spectrophotometer (CECIL CE7400S, London, United Kingdom) at 247 nm. The percentage drug content was calculated as follows [30]:

$$\%\;drug\;content=\frac{Actual\;Drug\;Loading}{The\;oratical\;Drug\;Loading}\times 100$$

##### In Vitro FBP Release from MPs

I In vitro FBP release from MPs (M3) was assessed using a dialysis membrane as described by Gajra, et al. [31] with some modification. About 10 mg of M3 containing ~2.5 mg of FBP was added to the cellulose acetate membrane (MWCO 10,000 Da) with 10 mL of PBS (pH 6.8). Both ends were sealed and placed in 200 mL of PBS at a 6.8 pH with continuous stirring at 50 rpm and 37 ± 2 °C to simulate oral cavity conditions. After 5 min, the pH of the media changed to 1.2 (0.1N HCl) for 2 h and then switched to pH 6.8 (PBS) to simulate intestinal conditions, and the study was continued for 48 h. At designated intervals, 5 mL of aliquot was withdrawn, filtered through a 0.45 µm syringe filter, and immediately analyzed by a UV-Visible spectrophotometer at 247 nm. Every time the withdrawn volume was replaced with an equal volume of a preheated fresh dissolution medium. All experiments were performed in triplicate. 

#### 2.2.5. Characterization of Composite ODF

##### Physical Properties

- Thickness

An electronic digital micrometer (resolution of 0.001 mm, SHAHE, Zhejiang, China) was used to measure the thickness of the composite films at five different points (center and edges) [30]. The mean thickness of the five films is reported in this study.

- Disintegration Time (DT)

The DT of the composite films was observed visually. The 6 cm^2^ film was placed in a Petri dish with 10 mL of a phosphate-buffered solution (pH 6.8) [30]. DT was considered when the film strip was completely disintegrated. The mean value of the observations is reported.

##### Mechanical Properties

- Folding endurance

Folding endurance is also called folding fortitude and provides evidence of the flexibility of the film. It was measured manually by holding the two sides of the film between the thumb and index finger of each hand. Then, the film was repetitively folded in the middle until the film fractured. The number of times the film was folded without breaking was reported as the folding endurance [30]. The mean of the triplicate observations is reported.

- Tensile strength

Tensile strength was measured using a Universal testing machine (INSTRON 3366-10KN, Instron^®^ GmbH, Darmstadt, Germany) equipped with 10 KN of the loaded cell. The film of 5 × 2 cm^2^ was clipped to the clamps; where one was fixed, the other was moveable. Both clamps were positioned at a distance of 3 mm. The film was pulled by the upper clamp at a rate of 5 mm min^−1^ until it broke to determine the tensile strength [32].

##### Solid State Characterization

The solid-state characteristics of pure drugs (FBP and RHCl), polymers (Lycoat^®^ RS780 and EC), and their formulations (F1 and F2) were accomplished using SEM, FTIR, XRD, DSC, and TGA, as described in Section Solid State Characterization of MPs.

##### Drug Contents

The drug content in F1 and F2 was evaluated by completely dissolving 6 cm^2^ ODF in ethanol, which was diluted with PBS pH 6.8. The content of RHCl and FBP was determined using a UV/Visible Spectrophotometer at 315 and 247 nm, respectively [33]. 

##### In Vitro Drug Release

The in vitro FBP release from F1 and F2 was accomplished as described in Section In Vitro FBP Release from MPs. For RHCl release from F2, samples from the first five minutes of release at pH 6.8 were analyzed at 315 nm. The release was performed in triplicate, and the mean was considered.

#### 2.2.6. In Vivo Studies

In vivo studies were conducted on Albino Wistar rats (150–250 g) obtained from an in–house animal facility. The Institutional Review Committee, Government College University Faisalabad Pakistan, approved all the protocols (Ref. No., GCUF/ERC/17, dated: 03-12-2021). The rats were housed in a controlled environment, i.e., 25 ± 1 °C, relative humidity of 60% ± 10%, and appropriate light and dark cycles. All animals were given standard food and water ad libitum. F1 and F2 were administered to animals and were equivalent to 5 mg/kg of FBP. Pure FBP was also administered at 5 mg/kg for comparison [34].

##### Anti-Inflammatory Activity

Carrageenan-induced rat paw edema was employed to investigate the anti-inflammatory activity. Albino Wistar rats weighing 150–200 g of either sex were divided into 5 groups (n = 3). The paw diameter of all the rats was determined using a digital Vernier caliper before giving any treatment or anti-inflammatory agent. Afterward, the first group received a 0.5% carboxymethyl cellulose (CMC) solution, the second received pure FBP, the third received M3, the fourth received F1, and the fifth received F2 orally. Pure FBP and M3 were suspended in 0.5% of a CMC solution given via a cannula. Composite films were cut into pieces and directly placed in the rat’s mouth using forceps. After one hour of treatment, acute inflammation was induced in the left hind paw via the intraplantar injection of 0.1 mL of freshly prepared 1% carrageenan solution in normal saline. The linear paw circumference was monitored for 10 h, and the percentage of inhibition was calculated as follows [35].

$$\%\;inhibition\;of\;paw\;edema=\frac{\left(Ct-C0\right)control-\left(Ct-C0\right)treated}{\left(Ct-C0\right)control}\times 100$$

where

Ct = left hind paw thickness (mm) at time t
C0 = left hind paw thickness (mm) before carrageenan injection
(Ct − C0) control = increase in paw size after carrageenan injection to control rats at time t
(Ct − C0) treated = increase in paw size after carrageenan injection to treated rats at time t.

##### Pro-Inflammatory Cytokines

For the quantification of TNF-α and IL-6 in the rat serum, blood samples were collected by a cardiac puncture from an anesthetized rat in an Eppendorf tube and kept for 15 min. Afterward, the blood was centrifuged at 10,000 rpm for 15 min. The serum was collected in Eppendorf tubes with the help of a micropipette and preserved at −20 °C for cytokine estimation. The rat-specific enzyme-linked immunosorbent assay (ELISA) kit was purchased from Elabscience^®^ (Wuhan, China) and used for the estimation of TNF-α and IL-6 as per the manufacturer’s instructions. The absorbance was detected at 450 nm with the ELISA plate reader [36].

##### In Vivo Evaluation of Gastroprotective Effect

Albino Wistar rats weighing 160–220 g of either sex were divided into 5 groups (n = 3). These groups were used to compare the ulcerogenic potential of pure FBP, M3, F1, and F2. Before administration, animals were fasted overnight with water ad libitum. The animals received 5 mg/kg of a pure drug or formulation containing an equivalent quantity of drugs Treatment was continued for five days, and the animals were sacrificed on the 5th day after dosing.

- Gastric lesion index (GLI)

Isolated stomachs were incised through a great curvature and washed with normal saline. After a macroscopic examination of the gastric tissues, ulcerative lesions were measured by a Vernier caliper. An arbitrary score (AS) was given to the ulcer like “0” for no ulcer/lesion, “0.5” for one or more ulcers of length < 1 mm, “1” for ulcers/lesions of length 1–2 mm, and “2” for ulcers/lesions with length > 2 mm. This arbitrary score was multiplied by the number of lesions to determine the lesion index [37].

$$Gastric\;lesion\;index\left(GLI\right)=AS\;\times \;No.of\;lesion/ulcers$$

After that, the stomachs were then preserved in a 10% formalin solution for histopathological analysis.

- Histological evaluation

Stomach tissues from each group were sliced into thin sections, and slides were developed after treating them with a hematoxylin and eosin (H & E) stain. These slides were observed under an Accu-scope 3000-LED microscope at different resolutions [37].

## 3. Results and Discussion

### 3.1. Fabrication and Evaluation FBP Loaded MPs

FBP-loaded EC MPs were successfully prepared using different polymer-to-drug ratios (Table 1). The organic phase was injected via an infusion pump (Figure 1a), which ensured droplet formation within an optimal size range. Furthermore, this automation reduced the chance of errors, enhanced drug loading, and the loss of ingredients [38]. Furthermore, evaporation continued for one hour and was dried overnight to bring the DCM within the permissible limits. DCM is a volatile liquid with a boiling point of 40 °C and hence could be easily evaporated at the provided conditions [39]. It was observed that M1 and M2 contained bigger, irregularly shaped microparticles with agglomerates. At low polymer-to-drug ratios, drug molecules were also bound to the surface of MPs and were responsible for large particles and agglomerates [17]. Thus, these two formulations were deemed inappropriate for dispersion in composite films. On the other hand, bulk density, tapped density, compressibility index, Hausner’s ratio, and an angle of repose of M3 were 0.16 g/mL, 0.18 g/mL, 11.11%, 1.13 and 23°, respectively, were within the permissible limits and indicated the free-flowing nature of microparticles. Therefore, M3 was chosen for further characterization and loading in the composite films. 

#### 3.1.1. Solid State Characterization of FBP Loaded MPs

**Figure 2:**
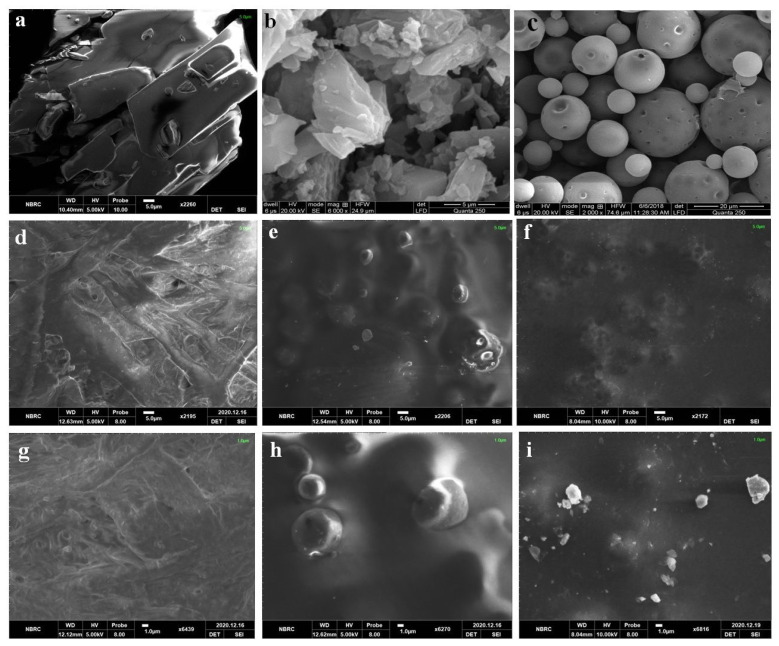
SEM of (**a**) FBP, (**b**) RHCl, (**c**) M3, (**d**,**g**) L15, (**e**,**h**) F1, and (**f**,**i**) F2. See Supplementary Figure S1 for optical images.

**Figure 3:**
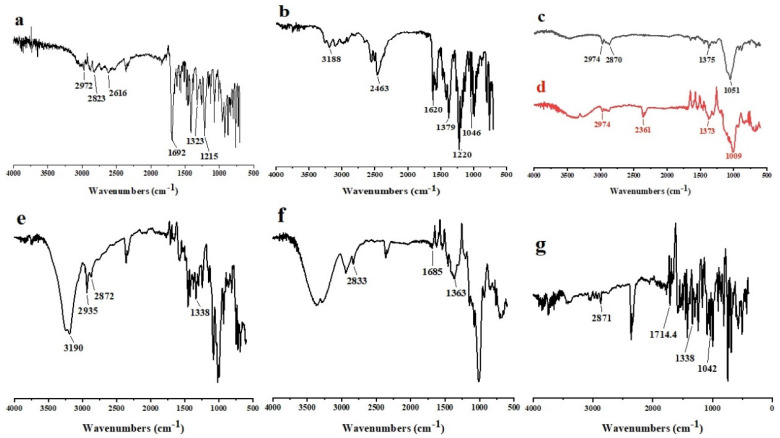
FTIR Spectra of (**a**) FBP, (**b**) RHCl, (**c**) EC, (**d**) Lycoat^®^ RS780, (**e**) M3, (**f**) F1, and (**g**) F2.

**Figure 4:**
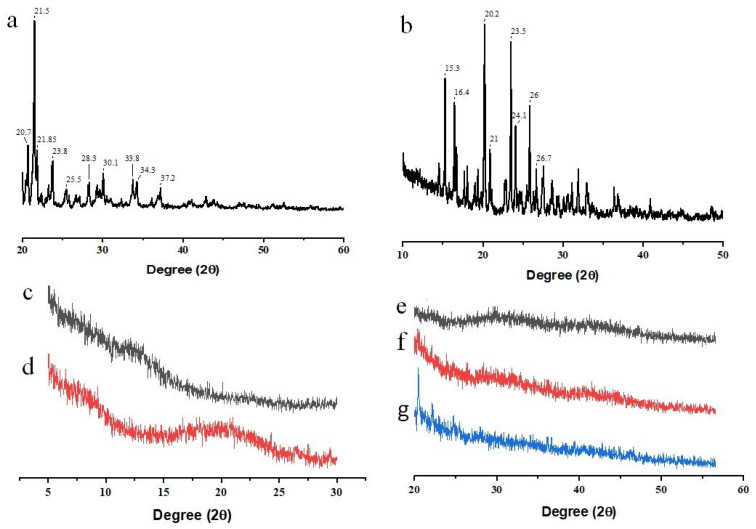
XRD of (**a**) FBP, (**b**) RHCl, (**c**) EC, (**d**) Lycoat^®^ RS780, (**e**) M3, (**f**) F1, and (**g**) F2.

**Figure 5:**
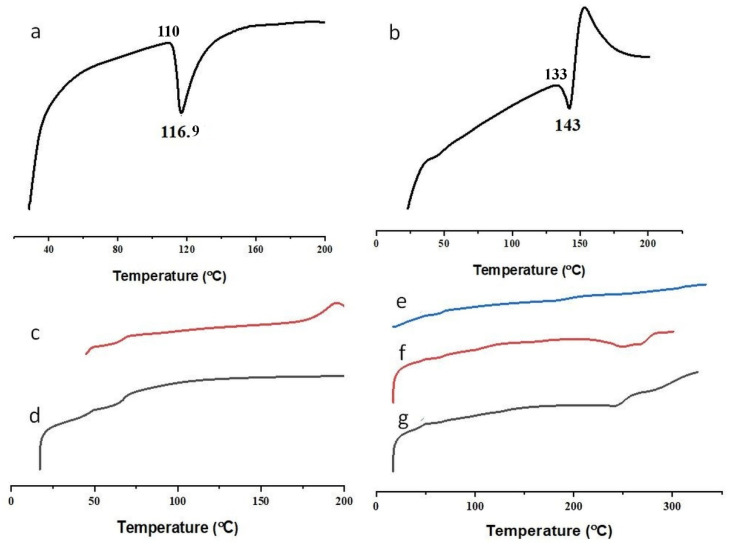
DSC of (**a**) FBP, (**b**) RHCl, (**c**) EC, (**d**) Lycoat^®^ RS780, (**e**) M3, (**f**) F1, and (**g**) F2. The onset temperature of FBP and RHCl was 110 and 133 °C, respectively.

**Figure 6:**
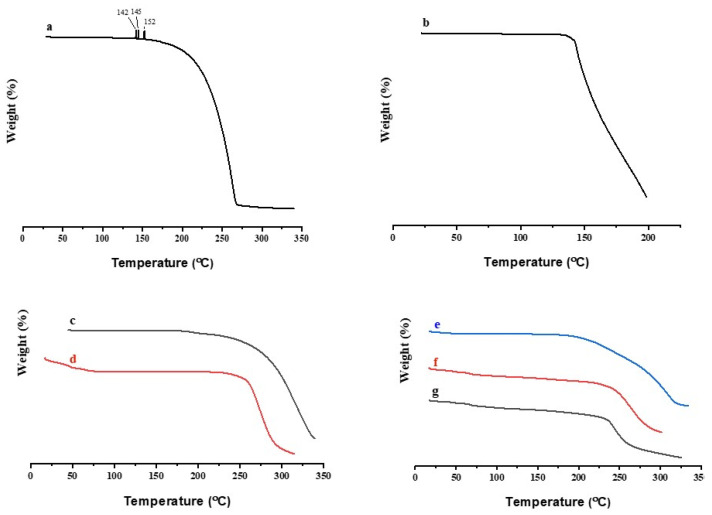
TGA of (**a**) FBP, (**b**) RHCl, (**c**) EC, (**d**) Lycoat^®^ RS780, (**e**) M3, (**f**) F1, and (**g**) F2.

SEM micrograph showed crystals of FBP (Figure 2a), and M3 depicted uniform-size spherical particles with a smooth surface (Figure 2c). However, these particles also showed small indents that could be due to water desorption during the drying process. Barbosa, et al. [40] reported spherical and uniform-size 5-fluorouracil-containing chitosan microspheres when prepared with a modified automatic drip (infusion) system. The FTIR spectra of FBP showed its characteristic peaks at 1215 cm^−1^, 1323 cm^−1^, and 1692 cm^−1^ for the fluoride, methyl, and carbonyl groups, respectively (Figure 3a). Furthermore, the vibrational band at 3500–2500 cm^−1^ represented characteristic hydrogen bonding in FBP [41]. The FTIR spectra of M3 showed major peaks of FBP (Figure 3e), thus indicating compatibility among the drug and carrier components. In XRD studies, pure FBP showed sharp peaks indicating its crystalline nature (Figure 4a), while pure EC displayed amorphous characteristics (Figure 4c). The XRD pattern of M3 showed diffused peaks (Figure 4e), which ed the amorphous nature of entrapped drugs. Thus, the dispersion of FBP at a molecular level coupled with a small particle size could enhance its dissolution and bioavailability, as observed previously [25]. The thermal changes in the formulation were evaluated by thermal analysis (DSC and TGA). The DSC of FBP showed a characteristic endothermic melting peak at 116.9 °C (Figure 4a), which is consistent with a previously published report [42]. The absence of the melting peak of FBP in M3 (Figure 5e) depicts the amorphous form of the encapsulated drug. TGA analysis confirmed the thermal stability of M3 over a tested temperature range (Figure 6e). 

#### 3.1.2. Drug Release from FBP Loaded MPs

**Figure 7:**
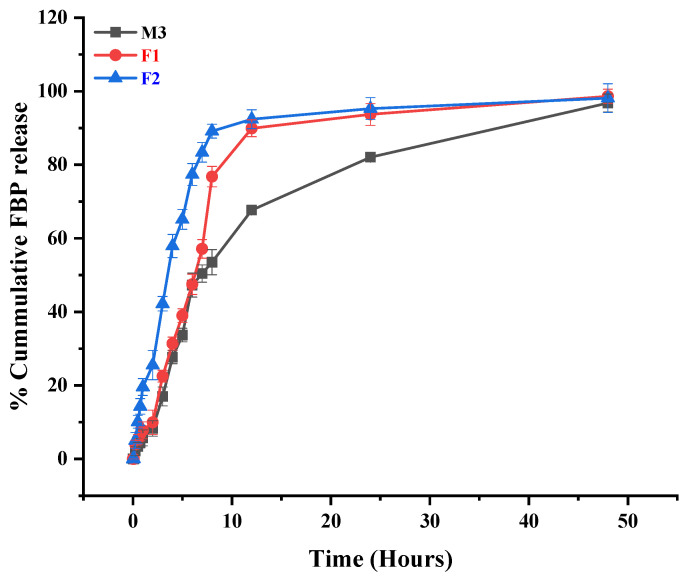
In vitro release profile of FBP from formulations M3, F1 and F2. The graph represents initial 5 min at 6.8 then at pH 1.2 for 2 h and finally up to 48 h at 6.8. Error bar represents ± SD (n = 3).

The percentage of the drug content for microparticles (M3) was 67.63%. The in vitro release profile of M3 was tested in simulated oral, gastric, and intestinal conditions (Figure 7). This formulation showed negligible FBP release at pH 6.8 during the first 5 min, and for the next 2 h, it released 2.14% of the entrapped drug at a pH of 1.2. When the medium was again switched to PBS (pH 6.8), it showed a rapid release initially and then gradually released the entrapped drug in a sustained release manner for up to 48 h. Although EC is insoluble at any pH, it undergoes pH-dependent swelling to some extent, which is higher in alkaline media than in acidic media. A similar drug release pattern was reported for fluconazole-loaded EC microspheres. They showed that, when media were switched from acid to alkaline, the swelling ratio increased from 2.11 to 4.86 within half an hour and was responsible for the initial burst release of the drug in alkaline media [43]. Other authors also documented similar observations for ethyl cellulose microspheres loaded with FBP [44] and metformin [25].

### 3.2. Fabrication and Evaluation of Blank ODF

The polymer concentration, plasticizer type, and super disintegrants play an important role in the development of ODFs. As Pearlitol Flash^®^ was employed as a super disintegrant at a constant concentration, only the role of the other two factors is discussed here. For ODFs, a minimum disintegration time and maximum flexibility were considered as important parameters. Here, the nature of the polymer affected the disintegration time, while the plasticizer provided desired flexibility [45]. Moreover, the concentration of the plasticizer also affected the disintegration time [46]. Initially, we screened different plasticizers (PG, PEG 400, and GLY) at various concentration levels (Table 1). After that, Lycoat^®^ RS780 was employed at different concentrations (Table 2) to select an optimum blank film. 

All the plasticizers were able to form peelable films with optimal flexibility when they were used at 10% *w/w* of the Lycoat^®^ RS780 (L3, L7, and L11 in Table 5). However, plasticizers at lower concentrations formed fragile and brittle films, while, at higher concentrations, ductile and sticky films were formed. This brittleness was observed due to an inadequate concentration of plasticizers, while stickiness was due to the excess oozing of the plasticizer from each film [27]. Furthermore, PG and PEG 400-containing films were excluded due to their low flexibility index, rough appearance, and high DT (Table 5). In comparison, films plasticized with glycerin were more flexible, durable, and supple to handle [27].

Among the various concentrations of the polymers tested, films fabricated with 10% *w/v* Lycoat^®^ RS780 exhibited superior characteristics, i.e., more flexible, transparent, and a minimum DT (Table 6). The literature also mentions that 10–15% of the polymer is sufficient to fabricate optimum ODFs [26]. After pre-formulation studies on seventeen different formulations, L15 was selected as the optimum blank formulation with a transparent appearance, DT around 20 s had a folding endurance > 300 times (Table 6). From here onwards, L15 was carried out for further work.

### 3.3. Fabrication of Composite ODF

For the development of composite films, optimized microparticles (M3) were dispersed in optimized Lycoat^®^ RS780 film (L15). After M3 loading, the composite film (F1) presented satisfactory parameters (i.e., transparent, flexible) except for the surface, which was slightly rough due to the presence of microparticles. The relative drug content was 90.89%. Lou, Liu, Qu, Hu, Brunson, Johnson and Almoazen [17] reported the development of composite ODF using Lycoat^®^ RS720. They dispersed chlorpheniramine maleate containing microparticles in ODF and possessed all the necessary characteristics of ODF with 99% drug loading.

After the successful incorporation of FBP-loaded MPs in the Lycoat^®^ RS780 based film (F1), we were interested to incorporate RHCl in the composite ODF (F2). The presence of RHCl in the formulation could reduce the side effects of FBP, as observed with continuous use of FBP while treating osteoarthritis, rheumatoid arthritis, mild to moderate dental pain, menstrual cramps, and muscle aches. To the best of our knowledge, this is our first attempt to develop composite films containing two drugs that could target two different segments of GIT, i.e., RHCl in an oral cavity and the majority of FBP encapsulated in microparticles, for release into the small intestine. Our results reveal that both drugs were successfully incorporated in composite ODF. F2 was durable and flexible, whereas the relative drug content for RHCl was 97.3% and 90% for FBP. The initial attributes of composite films indicated the suitability of the method adopted for the incorporation of FBP microparticles and RHCl in ODF.

### 3.4. Physical Properties of Composite ODF

#### 3.4.1. Thickness

The films’ thickness was directly related to the quantity of the polymer and drug. Its uniform thickness ensured dose accuracy. Moreover, ODFs should lie in an optimum thickness range, i.e., 50–200 µm [19,47]. If films are too thin, they break during peeling, while too-thick films disintegrate slowly [48] and fail to release the encapsulated drug immediately. Moreover, the suitable thickness of ODF is also necessary for comfortable application in patients. Our developed composite films fall within an optimum range, as mentioned in Table 7. 

#### 3.4.2. In Vitro DT

During testing, F1 and F2 disintegrated quickly, i.e., within 16 s (Table 7), which is suitable for an oral fast-disintegrating delivery system. According to CDER guidelines, the DT should be ≤30 s for fast-disintegrating tablets and is equally applicable to fast-disintegrating oral films [49]. Furthermore, as per European Pharmacopeia specifications, ODF are expected to disintegrate within 180 s when placed on the tongue, while FDA sets a time frame of 30 s [50]. The DT of F1 was lower than the blank film (L15), which was possibly due to the presence of hydrophobic ethyl cellulose microparticles, which acted as a disintegrating agent and affected the integrity of ODF. Similar observations were reported for ODF containing water-insoluble nanocrystals of meloxicam [51]. Moreover, the DT of F2 was lower than F1, which could be attributed to the presence of freely water-soluble RHCl that further facilitated disintegration.

### 3.5. Mechanical Properties of Composite ODF

#### 3.5.1. Folding Endurance

The folding endurance of ODF indicated flexibility and strength to withstand handling and transportation. The higher the value of folding endurance, the higher the mechanical strength of the film and vice versa [52]. For developed composite ODFs, folding endurance was greater than 300 (Table 7), indicating their flexibility and toughness. In composite films, Lycoat^®^ RS780 imparted suitable mechanical strength, while the plasticizer provided the required flexibility. According to the literature, folding endurance ≥ 300 indicated excellent flexibility [53]. 

#### 3.5.2. Tensile Strength

Tensile strength is important for manufacturing as well as for the ease of handling of oral films [47]. An ideal film should have moderate tensile strength to endure the force or stress experienced during manufacturing, handling, packaging, and transportation. Furthermore, too rigid films also give a bad mouthfeel [54]. The tensile strength of the composite films (Table 7) lie in a moderate range to endure all possible stresses. For F2, tensile strength decreased slightly. It could be observed that when additional components were added to the developed films, their structural uniformity was affected which led to a reduction in tensile strength. For example, a reduction in tensile strength was recorded when α-tocopherol nanocapsules were incorporated in carboxymethyl cellulose films [55], clove oil was incorporated in films of HPMC [56] and aripiprazole-poloxamer^®^407 solid dispersion was dispersed at a higher concentration in Kollicoat^®^ based ODF [10].

### 3.6. Solid State Characterization of Composite ODF

#### 3.6.1. SEM

We used SEM micrographs of FBP, RHCl, M3, L15, F1, and F2 (Figure 2) to observe the respective microstructures. Under SEM, FBP and RHCl presented a crystalline nature. Drug crystals were not observed in their respective formulations (M3, F1, and F2), indicating their successful encapsulation. The surface of the blank film (L15) was rough with wrinkles. In composite films, MPs were well dispersed (F1 and F2), indicating their successful incorporation and their surface was much smoother. Moreover, the addition of RHCl did not affect the surface characteristics. Similar observations are reported for the apioca starch-based film, where the neat starch film surface was very rough but became smoother by raising the concentration of chitosan [57].

#### 3.6.2. FTIR

FTIR spectra provided evidence about the functional group of drugs as well as a possible drug carrier interaction. The FTIR spectrum of pure drugs (FBP and RHCl), polymers (Lycoat^®^ RS780, and EC), and optimized formulations (M3, F1, and F2) are shown in Figure 3. The FTIR spectra of FBP exhibited distinctive peaks corresponding to functional groups in the drug at 1215 cm^−1^ for the fluoride group (–F), 1323 cm^−1^ for methyl group (–CH_3_), and 1692 cm^−1^ for the carbonyl group (C=O) (Figure 3a). Moreover, the vibrational band located between 3500 and 2500 cm^−1^ represented hydrogen bonding [11]. The pure RHCl spectra (Figure 3b) displayed various characteristic stretching peaks at 1620 cm^−1^ for the C=N of nitronic acid, 1220 and 1379 cm^−1^ for NO_2_ (nitro group), 2463 cm^−1^ for NH (CH_3_)_2_ (dimethylamine group) and two distinct peaks for the primary amide group (NH) at 3188 and 3256 cm^−1^. A strong peak was observed at 1046 cm^−1^, which depicted the crystalline form II of RHCl as reported in previously published data [58]. For EC, a peak at 1051 cm^−1^ indicated the stretching of C–O–C, and the peak at 1375 cm^−1^ showed –CH bending. Further, the peaks at 2870 and 2974 cm^−1^ depicted the stretching of the –CH group (Figure 3c). The Lycoat^®^ RS780 IR spectra presented peaks at 1009 cm^−1^, 1375 cm^−1^, and 2974 cm^−1^, which were due to the C–O stretch, methyl –CH bend, and methyl –CH stretch, respectively. Further, a peak at 2361 cm^−1^ indicated the –CO2 group (Figure 3d). The characteristic peaks of FBP and RHCl were also detected in the spectrum of their respective formulations (M3, F1, and F2) (Figure 3e–g) with a slight shift in the absorption bands, which suggests an absence of interaction between the drug and composite ODF components.

#### 3.6.3. XRD

XRD provides useful information about the physical nature of the samples as amorphous or crystalline. Most of the active pharmaceutical ingredients (API) were in a crystalline form. Their XRD pattern can be used to identify and evaluate the physical nature of API after its encapsulation in the carrier system [59]. The diffractogram of pure FBP (Figure 4a) revealed its crystalline nature as it displayed strong peaks at the diffraction angle (2θ): 20.7, 21.5, 21.85, 23.8 and 30.1 with a PSD value of 554, 1565, 518, 428 and 329, respectively [42]. The pure RHCl (Figure 4b) also depicted a typical crystalline pattern with intense peaks at 2θ: 15.3, 16.4, 20.2, 21, 23.5, 24.1, and 26. These values are in accordance with previously published data by Herrada-Manchón, et al. [60]. EC and Lycoat^®^ RS780 (Figure 4c,d) showed diffused peaks, indicating the amorphous nature of the polymers [19,61]. However, the halo amorphous XRD pattern of the formulations (M3, F1, and F2) indicated the conversion of FBP and RHCl into their amorphous form in the respective formulations.

#### 3.6.4. DSC

The DSC was used to record phase transitions (crystallization or melting) and chemical reactions (decomposition) in a given sample under study [59]. The thermogram of FBP and RHCl exhibited endothermic peaks at 116.9 and 143 °C, respectively (Figure 5a,b), which corresponded to their melting points and confirmed the crystalline state of the drugs. The RHCl also showed an instant exothermic decomposition at 153.3 °C (Figure 5b) [62]. Moreover, the endothermic peak of RHCl revealed that it was present in a crystalline form II, as reported by Mirmehrabi, et al. [63]. The melting point of FBP and RHCl were, as reported in the literature [42,60]. The polymers employed in the development of composite films were amorphous in nature, as confirmed by the diffused peaks for EC (Figure 4c) [64] and Lycoat^®^ RS780 (Figure 5d) [65]. The endothermic peak of FBP was not visible in the MPs (M3) and composite films (F1 and F2) (Figure 5e–g). Similarly, we did not observe the thermal peak of RHCl in the film (F2) (Figure 5g). All developed formulations (M3, F1, and F2) were stable up to the temperature tested (Figure 5f,g). Similar results are reported by other authors who used FBP-loaded ethyl cellulose microspheres [44] and chlorpheniramine maleate MPs loaded in a Lycoat^®^ film [17], respectively.

#### 3.6.5. TGA

TGA was conducted to analyze the stability of the drug and polymers by the observing mass loss of these substances at a specific temperature, which is related to the degradation of these substances. In this study, the TGA of the pure drug (FBP and RHCl), polymers (EC and Lycoat^®^ RS780), and drug-laden formulations (M3, F1, and F2) were observed (Figure 6). The TGA graph shows how pure Lycoat^®^ RS780 and EC underwent single-stage thermal degradation after 250 °C [19,66]. The TGA curve of FBP reveals two characteristic mass losses. The first minor mass loss was between 140 and 155 °C, which could be attributed to the evaporation of water molecules such as the water of crystallization, while the second stage showed an abrupt mass loss between 200 and 300 °C due to the degradation of FBP [11]. RHCl showed a mass loss above 130 °C [67]. The TGA of drug-laden formulations (M3, F1, and F2) were stable. This thermal analysis (DSC and TGA) demonstrates the improved thermal stability of FBP and RHCl in the final formulations (M3, F1, and F2) compared to their pure form and the presence of a dual drug in the single-layer film, which did not deteriorate the thermal stability of the final formulation, i.e., F2.

### 3.7. In Vitro Drug Release from Composite ODF

**Figure 8:**
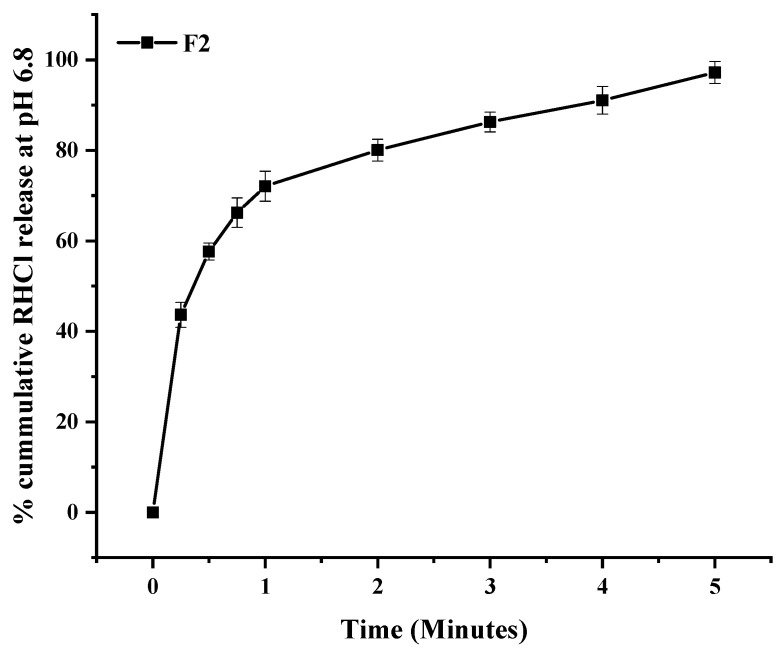
In vitro release profile of RHCl from formulation F2. Error bar represents ± SD (n = 3).

To assess the drug release pattern from F1 and F2, an in vitro release study was performed in simulated saliva, gastric and intestinal pH to mimic the GIT conditions using the beaker method. According to the literature, the acceptable dissolution volume ranges from 125 to 250 mL [13,68,69]. Figure 7 shows the release of FBP from M3, F1, and F2 in simulated GIT mediums, while Figure 8 shows the RHCl release from F2 in simulated salivary pH.

All the tested formulations showed a very negligible FBP release during the first 5 min at pH 6.8. That might be due to insufficient time to release the drug from the EC coating. Furthermore, as the medium was switched to pH 1.2 for 2 h, the formulations showed a negligible FBP release, i.e., 8.329% (M3) to 9.975% (F1), except for the F2 formulation, which showed a 25.52% release. Moreover, FBP release was considerably increased in the sustained release pattern as the pH of the medium was again switched to PBS (pH 6.8). The probable reason for this behavior was linked to the slight pH-dependent swelling of EC, which was higher in alkaline media [43]. Similar results were reported by Abdellatif, El Hamd, Ali and Saleh [44], and Raza, Javeria and Rashid [25], who developed sustained-release ethyl cellulose microspheres loaded with FBP and metformin, respectively. These composite films (F1 and F2) somehow exhibited a faster dissolution rate for FBP compared to FBP from MPs (M3). This was attributed to (1) the faster wetting of the microparticle in the film (2) the presence of a plasticizer and super disintegrating agent, which could act as a solubilizing agent and cause the partial dissolution of microparticles as observed previously [33]. On the other hand, the F2 film showed a significantly higher cumulative % release of FBP compared to M3 and F1, which could be due to the presence of hydrophilic RHCl. As per the literature, RHCl was responsible for enhancing the solubility and bioavailability of the water-insoluble drug, i.e., diclofenac [70]. 

In a composite film (F2), the cumulative % release of RHCl was 97% within 5 min (Figure 7), which is in agreement with previous studies about drug release from fast-disintegrating oral films. For example, Ouda, Dahmash, Alyami and Iyire [6]) reported 100% ibuprofen release within five minutes from the HPMC-based oral fast-disintegrating film. Another study showed that maximum chlorpheniramine maleate was released within 5 min from Lycoat^®^ RS780-based ODF [19].

### 3.8. In Vivo Studies

#### 3.8.1. Anti-Inflammatory Activity

**Figure 9:**
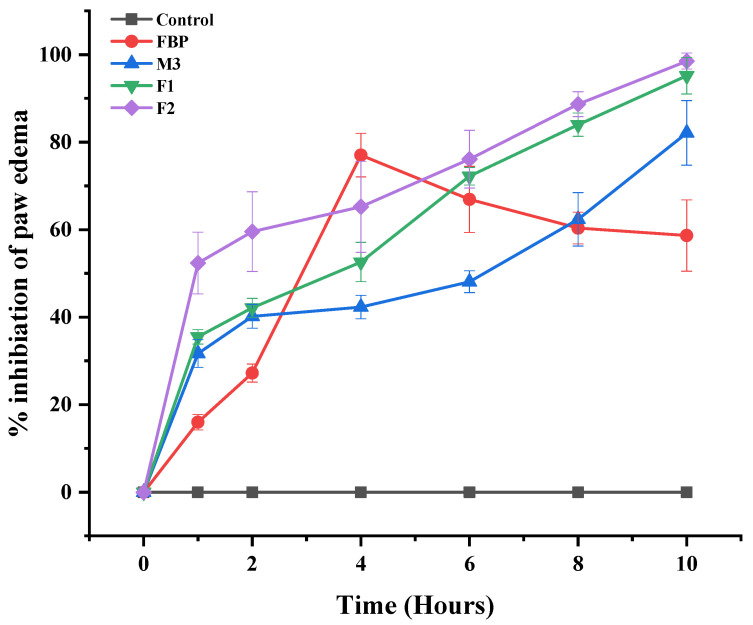
Percentage inhibition of paw edema after the administration of pure FBP and synthesized formulations (M3, F1 and F2). Error bar represents ± SD (n = 3).

To evaluate the anti-inflammatory potential of the proposed formulations (M3, F1, and F2), the carrageenan-induced rat paw edema model was employed. The anti-inflammatory activity of the synthesized formulation (M3, F1, and F2) was compared with the control (non-treated) and standard (pure FBP) groups, as shown in Figure 9. The pure FBP showed an inhibitory effect on paw edema for up to 4 h, after which it gradually declined. On the other hand, synthesized formulations (M3, F1, and F2) showed better inhibition of paw edema. This inhibitory effect gradually increased, and the maximum effect was observed at the 10th hour. Thus, these findings suggest that synthesized formulations (M3, F1, and F2) showed a sustained effect, and loading of MPs in composite oral films did not alter the release pattern and activity of FBP. When we compared all the formulations, the percentage inhibition of paw edema by F2 was relatively higher at all points. This revealed that the presence of RHCl in the F2 formulation did not suppress the anti-inflammatory activity of FBP; rather, it enhanced the inhibition rate by enhancing the solubility and bioavailability of FBP. In one study, flurbiprofen containing hyaluronic acid-coated bovine serum albumin nanoparticles showed improved therapeutic efficacy by reducing joint swelling when tested in an arthritis-induced rat model [71].

#### 3.8.2. Detection of Pro-Inflammatory Cytokines

**Figure 10:**
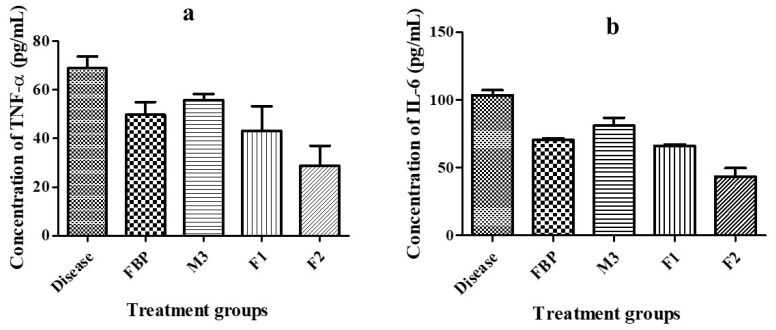
Rat serum level of (**a**) TNF-α and (**b**) IL-6 in the carrageenan-induced paw edema model. Error bar represents ± SD (n = 3).

Pro-inflammatory cytokines TNF-α and IL-6 play a pivotal role in the inflammation process. Therefore, serum levels of TNF-α and IL-6 in carrageenan-induced paw edema rat models were quantified by ELISA. We calculated their levels after 24 h of the treatment with pure FBP and optimized formulations (M3, F1, and F2). Figure 10a,b depicts how the TNF-α and IL-6 levels in the serum of the treated group were markedly reduced in contrast to the diseased group. The group treated with F2 induced a significant reduction in bothTNF-α and IL-6 levels in contrast to other treated groups. That might be due to the presence of the H_2_ receptor antagonist (Ranitidine HCl); Li, et al. [72] previously described that cimetidine, a well-known H_2_ receptor antagonist, significantly decreased the level of TNF-α and IL-6 in the treated group when compared with the diseased (ulcer) and control groups. Therefore, the results suggest that F2 exhibited superior efficacy compared to pure FBP.

#### 3.8.3. In Vivo Evaluation of Gastroprotective Effect

##### Gastric Lesion Index (GLI)

**Figure 11:**
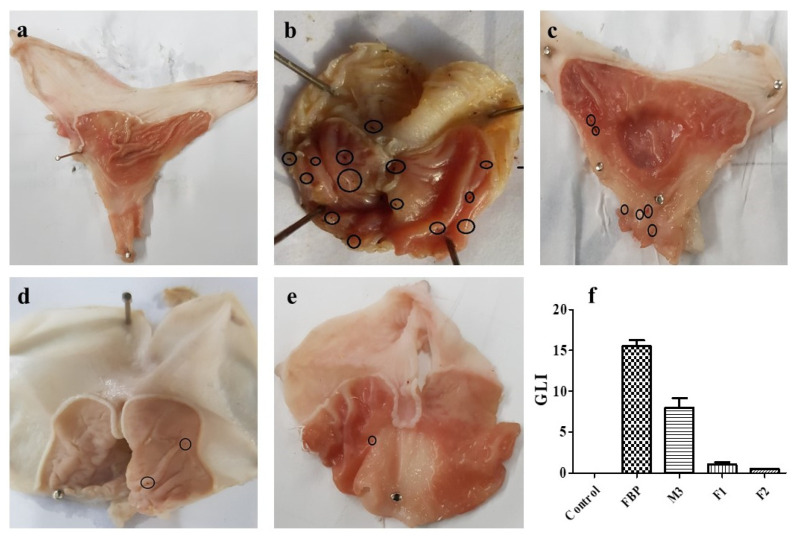
Photographs of rat stomachs from the (**a**) Control group, (**b**) Pure FBP-treated group, (**c**) M3-treated group, (**d**) F1-treated group, (**e**) F2-treated group, and the (**f**) Gastric lesion index. Error bar represents ± SD (n = 3). Black circles in figures indicate gastric ulcers. Explained in figure legends.

The gastroprotective effect of these formulations was determined by observing macroscopic lesions in the stomach mucosa of rats, as shown in Figure 11. The average score of gastric lesions was in the order of the pure FBP (15.5 ± 1.32) > M3 (8 ± 2) > F1 (1 ± 0.5) > F2 (0.5 ± 0) > control (0) (Figure 11f). It is well known that FBP causes gastric irritation, ulcers, erosions, and changes in mucosal permeability [73]; however, after encapsulation in the microparticles (M3), various gastric lesions were reduced compared to the pure drug (Figure 11b,c). Moreover, the macroscopic morphology, as shown in Figure 10d,e, clearly demonstrated that composite oral films (F1 and F2) markedly reduced the ulcer indices. Moreover, the proposed formulation containing RHCl (coded as F2) showed a lesion index close to zero. This was due to the presence of RHCl, which is an H_2_ receptor antagonist that inhibits the production of stomach acid and is commonly used to treat gastric lesions and ulcers [74]. 

##### Histological Evaluation

**Figure 12:**
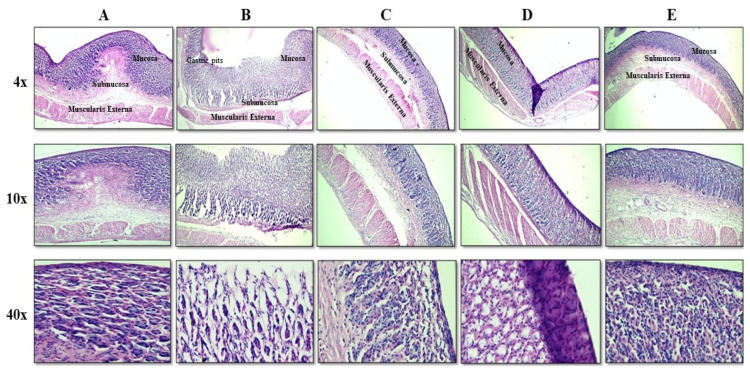
Histological images of stomachs taken at various resolution in different groups. (**A**) Control group, (**B**) Pure FBP-treated group, (**C**) M3-treated group, (**D**) F1-treated group, and (**E**) F2-treated group.

Figure 12 comprehensively demonstrates the histological changes in rat stomachs. The control group (Figure 12A) showed intact gastric mucosa without any visible erosion or inflammation, while the FBP-treated group (Figure 12B) showed a damaged gastric mucosal layer with focal surface erosion and inflammation in the deeper layer of the mucosa and early submucosa. There were no hemorrhagic areas and ulceration, or perforation. After the administration of micro formulation (M3), it was observed that gastric mucosa showed intact mucosal folds with inflammatory infiltration in the deeper layer of mucosa and early submucosa with focal superficial erosions (Figure 12C). Likewise, the gastric specimen of the group administered with a composite film F1 showed an intact mucosal layer with inflammatory infiltration in the upper mucosal layer (Figure 12D). Upon the administration of composite film F2, the gastric mucosa showed preserved mucosal folds and regular mucus-secreting glands lined along the epithelial cells. Moreover, there were no erosions or inflammatory infiltration (Figure 12E).

## 4. Conclusions

This innovative study demonstrates the development of composite ODF for the co-loading and sustained release of FBP MPs and plain RHCl through the solvent casting method. This strategy does not only avoid the undesirable effects of FBP on GIT but also helps avoid multiple tablets and multiple-dose intake. The SEM images of M3, F1, and F2 showed that microparticles (M3) were of even size and were uniformly distributed in the composite film (F1 and F2). Solid-state characterization suggested that co-loading was successfully achieved without any significant drug–polymer interaction. The in vitro release profile showed that encapsulated FBP was released in a controlled manner over 48 h, and plain RHCl was released immediately, i.e., within 5 min. Moreover, in vivo studies demonstrated that anti-inflammatory and gastroprotective effects were enhanced by the F2 formulation when compared with pure FBP and other purposed formulations (M3 and F1). Thus, we can comprehensively conclude that the fabricated composite ODF (F2) possesses optimum physicochemical and drug release properties. F2 effectively demonstrated its ability to overcome the adverse effects of FBP and could address the pitfalls of conventional drug delivery systems. In the future, developed ODF could reduce the dosage form burden and multiple doses to enhance patient compliance. 

## Figures and Tables

**Table 1:** Composition of microparticles.

Code	Organic Phase		Aqueous Phase	
	Polymer to Drug Ratio	DCM	Surfactant (PVA 1500)	Distilled Water
M1	1:1	20 mL	0.5% *w/v*	100 mL
M2	2:1			
M3	3:1			

**Table 2:** Selection of optimum plasticizer with constant concentration of Lycoat^®^ RS780 (*w/v* of total formulation).

Code	Lycoat^®^ RS780	Pearlitol Flash^®^	Plasticizer (% *w/w* of Polymer)			Water (mL)
	(% *w/v*)	% *w/w* of Polymer	PG	PEG 400	GLY	
L1	10	10	1	s		10
L2			5			
L3			10			
L4			15			
L5	10	10		1		10
L6				5		
L7				10		
L8				15		
L9	10	10			1	10
L10					5	
L11					10	
L12					15	

Each formulation contained the release modifier Pearlitol Flash^®^ Mannitol Starch (PF) % *w/w* of the polymer [27]. Here, PEG 400 stands for polyethylene glycol, PG for propylene glycol and GLY for glycerin.

**Table 3:** Selection of optimum concentration of Lycoat^®^ RS780 with constant concentration of plasticizer.

Code	Lycoat^®^ RS780 (%*w/v*)	Glycerin (%*w/w* of Polymer)	Pearlitol Flash^®^ (%*w/w* of Polymer)	Water (mL)
L13	1	10	10	10
L14	5			
L15	10			
L16	15			
L17	20			

**Table 4:** Composition of composite ODFs.

Code	Lycoat^®^ RS780 (g)	GLY (g)	Pearlitol Flash^®^ (g)	M3 (g)	RHCl (g)	Water (10 mL)
L15	1	0.1	0.1	-	-	10
F1	1	0.1	0.1	0.15	-	10
F2	1	0.1	0.1	0.15	0.15	10

**Table 5:** Physical parameters and the DT of blank films with different plasticizers at a constant concentration of Lycoat^®^ RS780.

Code	Appearance	Folding Endurance	DT (Seconds)
L1	No film form	–	–
L2	Fragile and Brittle, Translucent	137	111 ± 0.032
L3	Flexible, Durable, Translucent	198	164 ± 0.135
L4	Ductile and Sticky, Translucent	52	210 ± 0.318
L5	No Film Form	–	–
L6	Fragile and Brittle, Rough Surface	76	30 ± 0.177
L7	Flexible, Durable, Rough Surface	302	99.30 ± 0.337
L8	Ductile and very Sticky	Not performed	190 ± 0.178
L9	No Film Form	–	–
L10	Fragile and Brittle, Smooth, Transparent	>300	20 ± 0.163
L11	Flexible, Durable, Smooth, Transparent	300	58.59 ± 0.396
L12	Ductile and Sticky	100	70 ± 0.19

**Table 6:** Physical parameters and DT of blank films with a different Lycoat^®^ RS780 concentration at a constant plasticizer (GLY) concentration.

Code	Appearance	Folding Endurance	DT (Seconds)
L13	No film formation	-	-
L14	Very thin film formed, break during peeling	-	-
L15	Smooth, flexible, durable, transparent and easily peelable film formed	>300	20 ± 1.52
L16	Thick, smooth, transparent and brittle in nature	180	60 ± 1.83
L17	Thick, smooth, transparent and brittle in nature	110	70.03 ± 1.29

**Table 7:** Physical and mechanical properties of composite films.

Code	Thickness (µm)	Folding Endurance	Tensile Strength (N/mm^2^)	DT (Seconds)
F1	71 ± 0.021	350	2.75 ± 0.25	15.63 ± 1.01 s
F2	75 ± 0.018	300	2.49 ± 0.19	15.01 ± 0.13 s

## Data Availability

Most of the data are presented in the article. However, the raw or processed data that were required to reproduce these findings cannot be shared at this time due to technical or time limitations.

## Supplementary Materials

The following supporting information can be downloaded at: https://www.mdpi.com/article/10.3390/pharmaceutics15071987/s1, Figure S1: Apparent image of (a) M3, (b) L15, (c) F1, and (d) F2; Figure S2: Optical images showing DT of F1.
