# Supplementary material for: In Vitro and In Vivo Evaluation of Composite Oral Fast Disintegrating Film: An Innovative Strategy for the Codelivery of Ranitidine HCl and Flurbiprofen
Source: Pharmaceutics. 2023 Jul 20;15(7):1987. doi: 10.3390/pharmaceutics15071987 (PMC10383263; doi:10.3390/pharmaceutics15071987)
Supplement: Supplementary file 1 [file pharmaceutics-15-01987-s001.zip › pharmaceutics-2440631-supplementary.pdf]

In Vitro and In Vivo Evaluation of Composite Oral Fast Disintegrating Film: An Innovative Strategy for the Codelivery of Ranitidine HCl and Flurbiprofen

**Aisha Rashid [1], Syed Haroon Khalid [1], Muhammad Irfan [1], Sajid Asghar [1], Waleed Y. Rizg [2,3], Fahad Y. Sabei [4], Eman Alfayez [5], Hanaa Alkharobi [5], Awaji Y. Safhi [4], Khaled M. Hosny [3], Muhammad Sohail Arshad [6]
and Ikram Ullah Khan [1,*]**

[1] Department of Pharmaceutics, Faculty of Pharmaceutical Sciences, Government College University Faisalabad, Faisalabad 38000, Pakistan; draisharashid@gmail.com (A.R.); haroonkhalid80@gmail.com (S.H.K.); manipharma1@gmail.com (M.I.); sajuhappa@gmail.com (S.A.)
[2] Center of Innovation in Personalized Medicine (CIPM), 3D Bioprinting Unit, King Abdulaziz University, Jeddah 21589, Saudi Arabia; wrizq@kau.edu.sa
[3] Department of Pharmaceutics, Faculty of Pharmacy, King Abdulaziz University, Jeddah 21589, Saudi Arabia; kmhomar@kau.edu.sa
[4] Department of Pharmaceutics, College of Pharmacy, Jazan University, Jazan 45142, Saudi Arabia; fsabei@jazanu.edu.sa (F.Y.S.); asafhi@jazanu.edu.sa (A.Y.S.)
[5] Department of Oral Biology, Faculty of Dentistry, King Abdulaziz University, Jeddah 80209 , Saudi Arabia; ealfayez@kau.edu.sa (E.A.); halkharobi@kau.edu.sa (H.A.)
[6] Department of Pharmaceutics, Faculty of Pharmacy, Bahauddin Zakariya University, Multan 60800, Pakistan; sohailarshad@bzu.edu.pk
* Correspondence: ikramglt@gmail.com

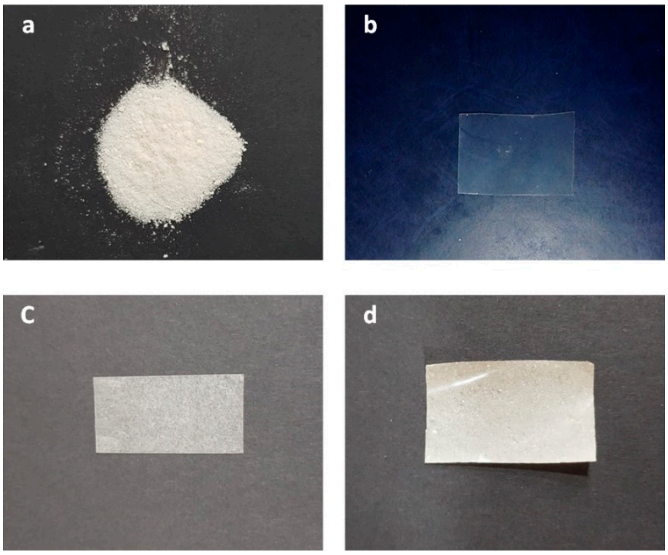

Figure S1. Apparent image of (a) M3, (b) L15, (c) F1, and (d) F2.

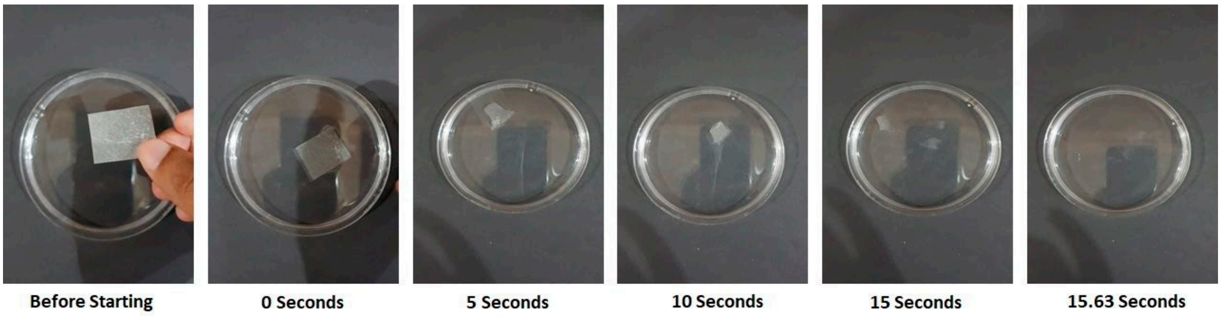

Figure S2. Optical images showing DT of F1.
